# A Case of Influenza A (H3N2) Complicated by Community-Acquired Pneumonia and Death in a Young Healthy Adult during the 2013–2014 Season

**DOI:** 10.3389/fpubh.2017.00001

**Published:** 2017-02-08

**Authors:** Lauren F. Collins, Benjamin D. Anderson, Gregory C. Gray

**Affiliations:** ^1^Department of Internal Medicine, Duke University Medical Center, Durham, NC, USA; ^2^Division of Infectious Diseases, School of Medicine and Global Health Institute, Duke University, Durham, NC, USA

**Keywords:** influenza A virus, *Staphylococcus aureus*, community-acquired pneumonia, influenza vaccine, neuraminidase inhibitors

## Abstract

With multiple available vaccines and antivirals, seasonal influenza A is typically a self-limited acutely debilitating illness in young healthy adults. Here, we illustrate unexpected morbidity and mortality in a relatively young and healthy patient seen at a large tertiary care academic medical center for seasonal influenza A (H3N2) complicated by community-acquired pneumonia, hypoxic respiratory failure, septic shock, and death.

## Background

Seasonal influenza remains a major public health concern given its association with increased morbidity and mortality in certain high-risk populations. Recent modeling by the Centers for Disease Control and Prevention (CDC) estimated that from 2010–2011 to 2015–2016, flu-related hospitalizations in the United States ranged from a low of 140,000 (during 2011–2012) to a high of 710,000 (during 2014–2015) (1). Both the CDC and the United States Advisory Committee on Immunization Practices recommend that all individuals 6 months of age and older should receive influenza vaccination, which is an effective intervention in reducing transmission. The highest priority populations for vaccination are those with disproportionate risk of influenza-associated complications, hospitalizations, and death, including children, adults ≥65 years of age, persons with chronic medical conditions, and those with an immunocompromised state, including pregnant women (2).

We describe the case of a young non-pregnant immunocompetent female with well-controlled asthma who presented in spring 2014 with influenza-associated community-acquired pneumonia (CAP) secondary to methicillin-resistant *Staphylococcus aureus* (MRSA) complicated by hypoxic respiratory failure, septic shock, and ultimately death.

## Clinical Case

A 28-year-old female with well-controlled asthma and bipolar disorder presented to the emergency department (ED) with 1 day of shortness of breath. She attempted management at home with albuterol nebulizer treatments; however, this provoked cough with blood-streaked sputum. The prior week, her younger daughter was ill with fever, rhinorrhea, dry cough, and myalgias. The patient recalled similar symptoms for 1–2 days; however, they resolved before significant dyspnea ensued. She visited her primary care physician (PCP) 2 months prior for a left naris abscess at the site of a nasal ring. A wound culture grew MRSA, and she was treated with 7 days of doxycycline with clinical improvement. She declined influenza vaccination for the 2013–2014 season.

**Figure 1:**
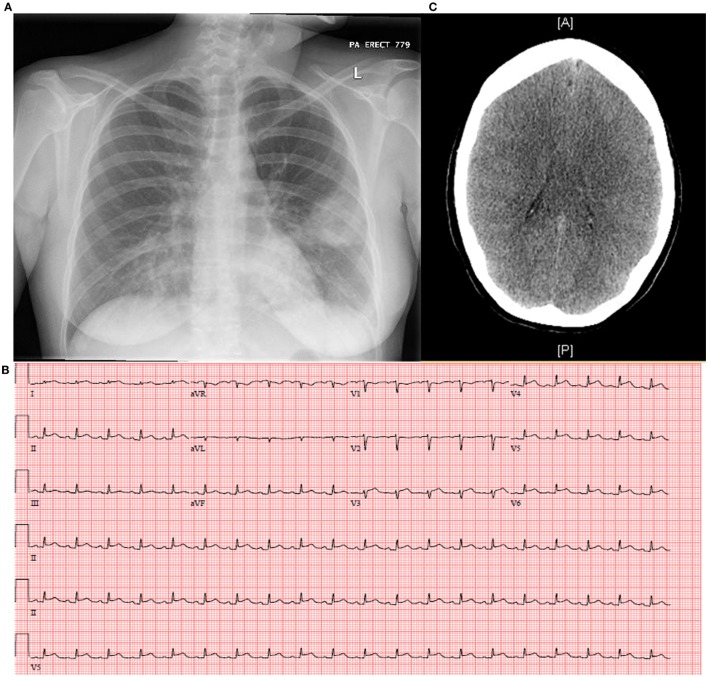
**Complications of seasonal influenza A may include (A) secondary bacterial pneumonia as demonstrated by the left mid-lung homogenous opacity on chest X-radiography, (B) myopericarditis as shown classically by electrocardiogram with diffuse ST elevations and PR depressions, and (C) diffuse cerebral edema not excluding viral encephalitis as revealed by computed tomography of the brain**.

On initial presentation to the ED in April 2014, the patient had a temperature of 37.1°C, heart rate of 140 beats per minute, blood pressure of 114/66, and respiratory rate of 18 breaths per minute with an oxygen saturation of 98% on room air. She was ill-appearing in mild respiratory distress with left lower lung field crackles, tachycardia without murmurs, and bilateral lower extremity pitting edema. Laboratory studies revealed pancytopenia with a total white blood cell count of 0.9 × 10^3^ cells/μl with left shift (58% polymorphonuclear leukocytes, 14% bandemia, 11% lymphocytes, 13% monocytes), hemoglobin of 11.6 g/dl, and platelet count of 110 × 10^3^ cells/μl. Her chemistry panel demonstrated normal kidney and liver function, with the exception of hypoalbuminemia. Urinalysis showed concentration (specific gravity of 1.041) with proteinuria and hyaline casts. Qualitative urine pregnancy test was negative and serum antibodies against human immunodeficiency virus (HIV)-1 and -2 were not detected. Chest radiography revealed a homogenous left mid-lung infiltrate with possible cavitation (Figure 1A). Electrocardiogram (ECG) displayed sinus tachycardia without ST or T wave changes. She was started on ceftriaxone and azithromycin for CAP with plan for admission.

Her condition then rapidly deteriorated with development of fever to 38.5°C, hemodynamic instability (sinus tachycardia to 160 beats per minute, blood pressure of 82/53), and respiratory decompensation (tachypnea of 62 breaths per minute, oxygen saturation of 85% on room air, use of accessory muscles, inability to speak). She required emergent intubation for hypoxic respiratory failure and initiation of vasopressor therapy in addition to aggressive fluid resuscitation for septic shock secondary to pneumonia. Her antibiotic regimen was broadened to vancomycin, piperacillin-tazobactam, azithromycin, and she was admitted to the medical intensive care unit.

Given the degree of her shock with significant lactic acidosis, she was additionally supported with stress dose steroids, transfusion of packed red blood cells, and a bicarbonate infusion. A computed tomography (CT) angiography of the chest though negative for pulmonary embolism revealed extensive dense consolidative airspace opacities within both lower lung lobes, demonstrating progression from the chest film earlier that day and suggestive of a necrotizing process. Clindamycin was added for antitoxin effect and doxycycline for possible rickettsial infection. Gynecology was consulted to evaluate for pelvic source of infection given reported intrauterine device and risk of toxic shock syndrome. Given worsening fever to 39.8°C and elevated creatine kinase, psychotropic medications for bipolar disorder were discontinued, given possibility of neuroleptic malignant syndrome.

Extended respiratory viral panels obtained on the day of admission by nasopharyngeal swab and bronchoalveolar lavage (BAL) were reported on hospital day 2–3 as positive for influenza A, subtype H3N2. Additionally, the BAL gram stain and culture returned as 4+ Gram-positive cocci in clusters, which speciated to MRSA. These new findings prompted urgent initiation of oseltamivir, and all other antibiotics were discontinued except for vancomycin on hospital day 3. Inability to wean vasopressors prompted investigation of cardiac function which unveiled myopericarditis: ECG with diffuse ST elevations and PR depressions most significantly in the late precordial leads to reciprocal changes in aVR (Figure 1B) and peak troponin T of 4.07 ng/ml, and transthoracic echocardiogram (TTE) disclosed severe global left ventricular hypokinesis with an ejection fraction of 25% and right ventricular dysfunction.

A number of other complications evolved, including acute renal failure, mild hepatitis, worsening pancytopenia, coagulopathy, and acute respiratory distress syndrome (ARDS) with development of bilateral pleural effusions requiring chest tube placement on the left. Despite discontinuation of sedation, her neurologic examination remained poor and CT brain obtained on hospital day 5 disclosed generalized cerebral edema with decreased differentiation of the gray–white matter junction (Figure 1C). Per consultation with neurology and neurosurgery, she most likely suffered anoxic brain injury secondary to cardiopulmonary collapse; however, influenza encephalitis could not be excluded. Lumbar puncture (LP) could not be safely performed, given severe illness with clinical instability. An extra-ventricular drain was placed to monitor intracranial pressure, hypertonic saline administered, and a cooling protocol enacted. Electroencephalogram showed diffuse slowing of brain waves 48 h later. A family meeting was held and the decision was made to withdraw all life support. She expired on hospital day 7.

## Methods

Viral RNA was extracted from amplified virus from our patient’s BAL specimen in MDCK cells using the QIAamp Viral RNA Mini Kit (QIAGEN, Inc., Valencia, CA, USA) and molecularly tested using the CDC Real-time RT-PCR (rRT-PCR) protocol for detection and characterization of influenza A (matrix), seasonal H1, seasonal H3, pandemic H1, H5, and H7. A two-step RT-PCR reaction was then performed using the same extracted viral RNA and universal primer sets targeting all eight influenza virus gene segments (3) and resolved on a 1% agarose gel stained with ethidium bromide. Amplified product was purified and submitted to a local sequencing company (Eton Bioscience, Inc., Raleigh, NC, USA). Sequence data were aligned and edited using BioEdit 7.1.9 (Ibis Biosciences, Carlsband, CA, USA) and then compared to the NCBI sequence database using the BLAST application.

## Results

Results from the CDC rRT-PCR assay demonstrated that our patient’s sample was positive for influenza A (matrix gene) and influenza A H3 subtype. Sequencing data from isolated gene segments (M, HA, NA, and NS) revealed a 97–98% identity to influenza A virus [A/North Carolina/13/2014(H3N2)].

## Discussion

This complex case of seasonal 2013–2014 influenza A, subtype H3N2, in a young immunocompetent non-pregnant female is unique in many ways. It reminds us that seasonal influenza can be deadly, when associated with pre-existing comorbidities and/or viral-related sequelae, such as bacterial coinfection. Mortality among influenza A infections often occurs in children or adults ≥65 years of age (4), those who are immunocompromised (5) and/or associated with bacterial coinfection (6). Associated mortality may disproportionately affect younger adults in seasons when particular virus types and subtypes are more lethal, such as the 2009 H1N1 influenza pandemic (7). Our patient differs from the higher risk clinical phenotype for influenza mortality, given her younger age in a non-pandemic season and lack of pregnancy or other immunocompromising state (e.g., HIV).

A retrospective study evaluating influenza-associated morbidity and mortality in women aged 15–44 found that in those who were considered high-risk—including having chronic lung disease—the estimated annual excess was 23 hospitalizations and deaths per 10,000 women compared to 4 per 10,000 in those without a high-risk condition (8). Comorbid asthma may have increased her risk of influenza complications; however, sequelae more commonly afflict patients requiring frequent corticosteroid use, which may predispose to delayed viral clearance (9) and increased susceptibility to bacterial pneumonia (6). Regardless of viral infection, asthma and frequent corticosteroid use have been identified as independent risk factors for bacterial CAP in young adults (10, 11), though the relevance of this in our patient is unknown, given she had moderate persistent asthma not requiring frequent systemic corticosteroid treatment.

Our patient’s course of H3N2 seasonal influenza A was complicated by secondary bacterial MRSA pneumonia resulting in hypoxic respiratory failure, septic shock, and ultimately, death. *Staphylococcus aureus* is the second most common pathogen after *Streptococcus pneumoniae* responsible for bacterial pneumonia superimposed on influenza infection (12). Incidence of *S. aureus* significantly increases in epidemic years (12), and recently, community-acquired MRSA (CA-MRSA) has been identified in outbreaks of severe pneumonia with a high mortality rate in young, otherwise healthy patients with influenza (13, 14). National data of CA-MRSA pneumonia during the 2006–2007 influenza season found the median age of cases as 16 years, 44% had no known pertinent medical history, and 51% died at a median of 4 days after symptom onset (13). This underscores the need for health-care providers during influenza season to be vigilant for the development of bacterial pneumonia in individuals without typical risk factors, such as increased age, comorbidities, or immunosuppression. Further, empiric CA-MRSA coverage should be considered in young adult patients with pneumonia, as this complication of influenza may be lethal.

Particularly striking about our patient’s case is the vast number of sequelae she developed in the setting of influenza coinfection with MRSA pneumonia, including ARDS, empyema, myopericarditis, kidney and liver injury, pancytopenia, coagulopathy, and cerebral edema with possible viral encephalitis. The pathophysiology of such conditions is most likely attributable to her overall degree of critical illness as well as bacterial coinfection with MRSA, but is also within the realm of influenza-associated morbidity (6, 15–19), especially in non-vaccinated individuals. It is not uncommon for pathology secondary to influenza to involve the muscle (17) and central nervous system (18), both sites of illness typically more common in children. Myopericarditis is a more rare complication of influenza (15, 16) and can be fatal, especially in the context of influenza B (19), prompting the need for early clinical detection as most patients require circulatory support and rapid escalation of care. Our patient demonstrated strong evidence of myositis and myopericarditis by substantial elevation in biomarkers of muscle injury (including those specific to the myocardium) and ECG/TTE findings, indicating globally decreased myocardial function likely due to inflammation, which could be viral-associated. Influenza encephalitis was suspected, though could not be confirmed by LP, given her state of critical illness.

Our patient declined the opportunity for influenza vaccine when evaluated by her PCP 2 months prior to presentation. The public health and individual benefits of influenza vaccination are clear, including promotion of herd immunity (20, 21), reduction in influenza-related hospitalizations, and decreased severity of illness (22). Additionally, a multicenter case–control study of adults and children hospitalized for CAP found patients with laboratory-confirmed influenza-associated pneumonia, compared with those with pneumonia not associated with influenza, had a lower odds of having received influenza vaccination [0.43, 95% confidence interval (CI), 0.28–0.68] (23). Isolation of our patient’s virus revealed 97–98% identity to Influenza A virus [A/North Carolina/13/2014(H3N2)]. According to data analyzed by the CDC, 95.3% of 426 influenza A (H3N2) viruses tested were characterized as Texas/50/2012-like, the influenza A (H3N2) component of the Northern Hemisphere quadrivalent and trivalent vaccines for the 2013–2014 season (24). End of the 2013–2014 influenza season estimates for vaccine effectiveness ranged from 39% (95% CI = −6 to 65%) for persons aged ≥65 years to 56% (CI = 37–69%) for persons aged 5–19 years (25). From October 2013 to May 2014, influenza vaccination resulted in an estimated 7.2 million (CI = 5.1–9.9) fewer illnesses, 3.1 million (CI = 2.1–4.4) fewer medically attended illnesses, and 90,068 (CI = 51,231–144,571) fewer hospitalizations associated with influenza (25). Interestingly, rates of influenza-related hospitalization for adults aged 20–64 were 1.3–5.5 times higher than during previous reported seasons (25–27). Taken together, this suggests that, had our patient been vaccinated against influenza in 2013–2014, her risk of developing laboratory-confirmed viral illness could have been reduced by 44–61%; however, likelihood of hospitalization may have been less affected, given the younger demographic requiring hospitalization in the 2013–2014 seasonal influenza.

Last, we query whether our patient’s burden of influenza-related morbidity and mortality could have been minimized if she were diagnosed with influenza earlier and/or empirically treated with antiviral therapy. Most studies of neuraminidase inhibitors show greatest benefit when therapy is initiated within the first 48 h of symptom onset (28). A large meta-analysis of randomized controlled trials evaluating oseltamivir compared to placebo found treatment of influenza in adults accelerates time to clinical symptom alleviation, reduces risk of lower respiratory tract complications, and decreases hospitalization (28). In that analysis, 838/1565 (53.5%) of patients were given oseltamivir >24 h after symptom onset, implying neuraminidase therapy may still be beneficial when administered later in the course of illness. This is further supported by data for 29,234 patients admitted to the hospital with pandemic influenza A H1N1 from 2009 to 2011, which found a reduction in mortality risk with neuraminidase inhibitor treatment—irrespective of timing—compared with no treatment; however, there was an increase in the mortality hazard rate with each day’s delay in therapy initiation up to day 5 compared to initiation within 2 days of symptom onset (29). In our patient’s case, nasopharyngeal- and BAL-sourced respiratory viral panels returned positive for influenza A (H3N2) on hospital day 2–3 prompting initiation of oseltamivir >48 h after her presentation with fever and dyspnea. Earlier recognition of influenza infection and prompt initiation of antiviral therapy may have prevented severe illness in this patient who ultimately succumbed to numerous complications, including death.

## Conclusion

Seasonal influenza can be fatal in young, otherwise healthy adults with the development of infectious sequelae such as bacterial pneumonia. Non-vaccinated individuals are at higher risk of influenza-related complications, including bacterial coinfection, which may drive clinical decompensation and even death. This specific case reminds us that influenza-related MRSA pneumonia is not uncommon in young, healthy adults, and early recognition of critical illness is crucial in order to promptly initiate more comprehensive management, including appropriate antiviral and antibiotic therapy, and care escalation as needed.

## Ethics Statement

This is a case report of a deceased patient; thus, written consent was not able to be obtained. The authors were granted a waiver of authorization of consent, approved by the Duke Institutional Review Board under protocol number Pro00070258. All information is anonymized as far as possible, and Duke University Health System standards were followed for publishing the case report of a deceased patient as reviewed by the Chief Compliance and Privacy Officer. The authors confirm that they have followed procedures in accordance with the Declaration of Helsinki.

## Author Contributions

LFC reviewed the patient’s medical chart, performed the literature review, and wrote the case report. BDA isolated and characterized the virus and contributed the methodology. GCG provided guidance and expertise in case review, laboratory work, and manuscript preparation.

## Conflict of Interest Statement

The authors declare that the research was conducted in the absence of any commercial or financial relationships that could be construed as a potential conflict of interest.
